# Genomic prediction in Persian walnut: Optimization levers according to genetic architecture of complex traits

**DOI:** 10.1002/tpg2.70047

**Published:** 2025-05-14

**Authors:** Anthony Bernard, Juliette Bénéjam, Morgane Roth, Fabrice Lheureux, Elisabeth Dirlewanger

**Affiliations:** ^1^ INRAE, University of Bordeaux, UMR BFP Villenave d'Ornon France; ^2^ INRAE, UR GAFL Montfavet France; ^3^ CTIFL, Centre opérationnel de Lanxade Prigonrieux France

## Abstract

Persian walnut (*Juglans regia* L.) is a widespread cultivated nut tree species in temperate regions. Advances in genomic tools, such as the high‐density Axiom *J. regia* 700K single nucleotide polymorphism (SNP) genotyping array, enable the exploration of genomic prediction (GP) for this crop. This study is the first to evaluate GP accuracy and several influencing factors in walnut for traits related to phenology and nut quality. A core‐collection of 170 accessions was phenotyped for 25 traits over 1 or 2 years. Highly heritable traits, such as budbreak date and female flowering date, were predicted with high accuracy (∼0.75) using ridge regression best linear unbiased prediction (rrBLUP). Three key factors influencing GP performance were examined: marker density, prediction model, and training set size. Selecting the top 1% of 364,275 SNPs based on their variance (∼3600 SNPs) was sufficient to achieve accurate predictions. Bayesian models slightly improved prediction accuracy for some traits when using this reduced SNP set, but rrBLUP provided robust results, balancing accuracy, simplicity, and computational efficiency. Training population size also influenced accuracy, with a subset comprising 50% of the population still yielding reliable predictions. Optimization of training set was assessed using coefficient of determination mean, prediction error variance mean, and mean relatedness (MeanRel) parameters, with MeanRel performing best for shell traits. However, incorporating SNPs identified in genome‐wide association study into the prediction models did not enhance accuracy. In summary, this study demonstrates the feasibility and potential of GPs for walnut breeding programs using a core‐collection, offering valuable insights for optimizing GP approaches in this crop.

## Core Ideas

- Genomic prediction (GP) accurately estimates traits in walnut using ridge regression best linear unbiased prediction (rrBLUP) and reduced single nucleotide polymorphism (SNP) set of 3600 SNPs.
- Marker density, training set size, and model significantly influence GP accuracy for phenology and nut traits.
- Bayesian models can enhance GP accuracy slightly, but rrBLUP offers robust, efficient performance overall.
- Training set optimization using mean relatedness improves GP accuracy for shell traits.

**Abbreviations**
BGLR: Bayesian generalized linear regression
CDmean: coefficient of determination mean
DArT: diversity arrays technology
G × E: genotype by environment
GP: genomic prediction
GS: genomic selection
GWAS: genome‐wide association study
INRAE: Institut National de Recherche pour l'Agriculture, l'Alimentation et l'Environnement
LASSO: least absolute shrinkage and selection operator
LD: linkage disequilibrium
MAS: marker‐assisted selection
MCMC: Markov Chain Monte Carlo
MeanRel: mean relatedness
PA: prediction accuracy
PEV: percentage of explained variance
PEVmean: prediction error variance mean
QTL: quantitative trait locus
rrBLUP: ridge regression best linear unbiased prediction
SNP: single nucleotide polymorphism
SSR: simple sequence repeat
STPGA: selection of training populations with a genetic algorithm
SVD: singular value decomposition

## 1 INTRODUCTION

Persian walnut (*Juglans regia* L.) is a monoecious tree belonging to the *Juglandaceae* family (Rehder, 1947) and the *Juglans* genus, which includes more than 20 species such as black walnut (*Juglans nigra*) used for its wood quality (Manning, 1978). *Juglans regia*, a diploid species with 2n = 2x = 32 chromosomes (Woodworth, 1930), originated from Central Asia before its dispersal to western areas, in Iran, the Caucasus, and eastern Turkey (Forde, 1975; Zeven & Zhukovsky, 1975), and was introduced into Europe and the Balkans before the last glacial period (Carrion & Sanchez‐Gomez, 1992; Pollegioni et al., 2017). Appreciated for its high‐quality kernel with nutritious value, Persian walnut is a widespread cultivated nut tree species in temperate regions, with a constantly increasing production that reached almost 3.9 million metric tons in 2022 (https://www.fao.org/faostat/), with major producers being China, the United States, Iran, and Turkey.

Walnut breeding programs started in the 20th century and have been or are currently led in California, France, China, Eastern Europe, and the Middle East with common goals: higher yield, easier shell cracking, kernels of higher quality, better adapted phenology, and better tolerance to diseases like anthracnose (Bernard et al., 2018; Cosmulescu & Botu, 2012). Breeding consisted traditionally in the selection of superior genotypes based on phenological and pomological observations, for instance in Iran (Ebrahimi et al., 2015; Ghasemi et al., 2012; Khadivi‐Khub et al., 2015) and in India (Sharma et al., 2014). Then, the emergence of molecular markers provided insights into the level of genetic diversity in plant material used for breeding (Bernard et al., 2018; Shah et al., 2021). The release of modern cultivars, such as Chandler in California in 1979 or Fernor in 1995 in France, represents a significant milestone in genetic improvement of this species with a long generation time and juvenile period. However, progress is still required to meet both producers and consumers current demand in the context of climate change strongly impacting phenological events, for example, in the United States and Europe, where earlier flowering increasingly exposes flowers and young fruits to late spring frosts (Bernard et al., 2019; Luedeling & Gassner, 2012).

The first walnut genome sequence (Martínez‐García et al., 2016) facilitated genomic studies, including the development of the high‐density Axio *J. regia* 700K single nucleotide polymorphism (SNP) genotyping array (Marrano, Martínez‐García et al., 2019). This tool enabled genome‐wide association studies (GWASs) for identifying numerous marker‐trait associations. Recently, 59 SNPs associated to calcium and phosphorus accumulation were identified using a panel of 154 accessions of genetic resources in Turkey and diversity arrays technology (DArT) genotyping (Akpunar et al., 2024), and 14 SNPs associated to shell thickness were identified using a panel of 101 accessions from 12 genotypes in China and Illumina resequencing (Wang et al., 2022). Phenology‐related traits were investigated using the Axiom SNP array and major loci at the beginning of chromosome 1 were detected for budbreak date (Bernard et al., 2020) and leafing date (Marrano, Sideli et al., 2019) in panels from the United States and France, respectively, whereas major loci at the beginning of chromosome 4 were observed for leafing date in a walnut collection in Turkey using DArT genotyping (Bükücü et al., 2020). Regarding fruit quality‐related traits, GWAS allowed the discovery of numerous SNPs associated with a large range of traits, such as nut weight, ease of cracking, nutritional composition, and pellicle pigment, using as well the Axiom SNP array (Arab et al., 2019; Bernard et al., 2021; Sideli et al., 2020). Even if the genomic regions involved in the variation of these traits of interest are not systematically consistent between the panels and the statistical models used, we now have a better knowledge of their overall genetic architecture in walnut: the traits of interest are primarily highly quantitative, indicating a polygenic inheritance.

The marker‐assisted selection (MAS) is increasingly adopted in walnut. It is implemented in the California breeding program in the framework of the “Walnut Genome and Molecular Breeding Project” (https://nealelab.ucdavis.edu/wgmb/); a kompetitive allele‐specific PCR marker was developed for the budbreak date using the peak SNP associated with chromosome 1 (Bernard et al., 2020), and Turkish simple sequence repeat (SSR) markers associated with leafing date (Kefayati et al., 2019) were validated in an Iranian population to identify late‐leafing genotypes (Fallah et al., 2024). However, to our knowledge, no study explored the effectiveness of genomic prediction (GP) in walnut. Genomic selection is a modern breeding strategy proposed by Meuwissen et al. (2001), which is particularly suitable for complex traits with polygenic inheritance (R2D2 Consortium et al., 2021). This strategy consists in the use genome‐wide SNP genotyping to predict breeding values, considering all markers effects (Makowsky et al., 2011). In genomic selection (GS), a training population is both phenotyped for the traits of interest and genotyped. The training population is used to train a prediction model, the latter being tested in a validation population only genotyped. The goal is to obtain high accuracy in the predictions of the phenotypes, as it was demonstrated in many fruit species such as apple for harvest date and fruit weight (Cazenave et al., 2022), apricot and citrus for fruit quality (Minamikawa et al., 2017; Nsibi et al., 2020), peach for soluble solids content (Hardner et al., 2022), and grapevine for yield, berry composition, phenology, and vigor (Brault et al., 2022).

Different factors are known to influence the prediction accuracy, including the marker density across the genome, the heritability and the genetic architecture of the traits, the size and the composition of the training set with regards to the validation test, and the prediction models (Alemu et al., 2024; Heslot et al., 2012; X. Liu et al., 2018). One of the first models proposed for GPs was the ridge regression (RR). This model is an equivalent to best linear unbiased predictions (BLUPs) for mixed models, allowing to fit the effects of a number of markers (*p*) largely exceeding the number of observations (*n*); a dilemma experienced by many plant breeders thanks to the evolution of genotyping methods (Hoerl & Kennard, 2000). Endelman (2011) developed the R package “ridge regression best linear unbiased prediction (rrBLUP)” that facilitated the use of RR for predictions. Because many models exist to implement these “large‐p‐with‐small‐n” regressions, based on various parametric and nonparametric shrinkage and variable selection procedures, Pérez and de los Campos (2014) developed the R package “Bayesian generalized linear regression (BGLR),” combining different models from Bayesian methods (Gianola et al., 2009). According to the genetic architecture of the trait considered, some models seem to perform better as it was observed in grapevine: RR is adapted for a trait underlined by many minor quantitative trait loci (QTLs), while least absolute shrinkage and selection operator (LASSO) is preferred for an oligogenic trait (Brault et al., 2021). Regarding training set design, its composition was first proved to influence the reliability of GP in maize inbreds (Rincent et al., 2012). Akdemir (2017) developed the R package “selection of training populations with a genetic algorithm (STPGA),” proposing different methods such as based on prediction error variance mean (PEVmean) and coefficient of determination mean (CDmean) (Laloë, 1993). CDmean was found to perform well to better predict firmness in apple (Roth et al., 2020), and criteria from mixed model theory such as CDmean and PEVmean perform well except in the case of large population structure (Isidro y Sánchez & Akdemir, 2021; Isidro y Sánchez et al., 2015). Prediction accuracy can also be improved when including markers identified in GWAS or QTLs as fixed‐effects in GP models, as it was shown in *Capsicum annuum* for capsaicinoid contents (G. W. Kim et al., 2022).

**Core Ideas**

- Genomic prediction (GP) accurately estimates traits in walnut using ridge regression best linear unbiased prediction (rrBLUP) and reduced single nucleotide polymorphism (SNP) set of 3600 SNPs.
- Marker density, training set size, and model significantly influence GP accuracy for phenology and nut traits.
- Bayesian models can enhance GP accuracy slightly, but rrBLUP offers robust, efficient performance overall.
- Training set optimization using mean relatedness improves GP accuracy for shell traits.

Here, we evaluated the potential of GP using the Institut National de Recherche pour l'Agriculture, l'Alimentation et l'Environnement (INRAE) walnut core‐collection of 170 accessions as a training set, resulting from sampling performed in 23 countries. The core‐collection was phenotyped for 25 traits related to phenology, such as flowering dates and fruit quality, including nut size, shell characteristics, and nutritional quality. It was genotyped using the high‐density Axiom *J. regia* 700K SNP array. We assessed several factors influencing the GP accuracy: number of SNPs, heritability of the trait, prediction model, and size of training set. Additionally, we tested different training set optimization criteria, and evaluated the impact of including GWAS‐identified SNP peaks as fixed‐effect cofactors. We hypothesize that different categories of traits will be best predicted by different types of models, and that both training set optimization and inclusion of GWAS results as cofactors will improve GP accuracy. Our objective is to compare the performance of MAS and GS based on performance metrics and provide insights for future walnut breeding programs.

## 2 MATERIALS AND METHODS

### 2.1 Plant material

The plant material used in this study is a core‐collection of 170 unique *J. regia* accessions originating worldwide and maintained at the INRAE *Prunus‐Juglans* Biological Resources Center (Roux‐Cuvelier et al., 2021; https://doi.org/10.17180/WN42‐3J20). Trees are grafted with one replicate by accession and located in the Fruit Experimental Unit of the INRAE, Toulenne, France (44°34′37.442″ N–0°16′51.48″ W, https://doi.org/10.17180/9ST1‐4J21), near Bordeaux. In Toulenne, the soil is deep fertile alluvium, homogeneous across the trees, and the climate is oceanic. The walnut germplasm collection results from large samplings performed between 1988 and 2000 in 23 countries including the European, North and South American, and Asian continents. The final core‐collection is based on a previous study integrating genetic diversity analyses using 13 SSR markers and the evaluation of phenotypic variability (Bernard et al., 2018). Information about origin of the accessions is available in the mentioned article.

### 2.2 Phenotyping for phenology and fruit quality, BLUPs calculation, and heritability

A total of 25 traits related to phenology and fruit quality were phenotyped over 1 year or two consecutive years during the period 2017–2019. The phenotyping was carried out in the framework of GWAS analyses already published by Bernard et al. (2020) for phenology‐related traits and Bernard et al. (2021) for fruit quality‐related traits. The 25 traits are categorized into five types of traits, namely, phenological, nut‐related, shell‐related, kernel‐related, and nutritional traits (Table S1). More precisely, four phenological traits were phenotyped on 155 accessions (dichogamy: PHENO_d, budbreak date: PHENO_bd, female flowering date: PHENO_ffd, male flowering date: PHENO_mfd); seven nut traits were phenotyped on 160 accessions (nut length: NUT_l, nut face diameter: NUT_fd, nut profile diameter: NUT_pd, nut volume: NUT_v, empty space volume: NUT_esv, nut filling ratio: NUT_fr, nut weight: NUT_w); seven shell traits were phenotyped on 160 accessions (shell sphericity: SHELL_s, shell rugosity: SHELL_r, shell volume: SHELL_v, shell thickness: SHELL_t, shell surface area: SHELL_sa, shell suture strength: SHELL_ss, shell face strength: SHELL_fs); five nutritional quality traits were phenotyped on 165 accessions (saturated fatty acids content: QUAL_sfa, monounsaturated fatty acids content: QUAL_mufa, polyunsaturated fatty acids content: QUAL_pufa, tocopherols content: QUAL_t, vitamin E activity: QUAL_vea); and two kernel traits were phenotyped on 160 accessions (kernel volume: KERNEL_v, kernel weight: KERNEL_w). Complete information about phenotyping methods and raw data are available in the mentioned articles.

The means of genotypic effects were obtained for each accession (one replication by accession) by adjusting for environmental factors using a mixed linear model to produce BLUPs. When using 2‐year data, the BLUPs were predicted by adjusting for year as a fixed effect as follows:

$$P_{ik}=\mu +Y_{i}+g_{k}+e_{ik},$$

where *P_ik_* is the observed phenotype of the *k*th accession in the *i*th year, *μ* is the mean value of the trait, *Y_i_* is the fixed effect of the *i*th year, *g_k_* is the random effect of the *k* genotype, and *e_ik_* is the residuals of the model. The BLUPs were performed using the “R” package “lme4” (Bates et al., 2015).

Based on the previous mixed linear models, broad‐sense heritability (*H* ^2^) of each trait was estimated using the variance components when 2‐year data are available:

$$H^{2}={\sigma^{2}}_{G}/\left[{\sigma^{2}}_{G}+\left({\sigma^{2}}_{\varepsilon }/n_{y}\right)\right],$$

where *σ* ^2^ *_G_* is the genotypic effect variance, *σ* ^2^ *_ε_* is the variance of residuals, and *n_y_* is the number of years = 2 (for 2017 and 2018 or 2018 and 2019, depending on the trait). A summary of the data available for each trait (minimum, maximum, and range) and broad‐sense heritabilities are given in Table S1.

### 2.3 SNP genotyping and quality control

The genomic DNAs were extracted from young leaves as described in Bernard et al. (2018). Accessions were genotyped using the Axiom *J. regia* 700K SNP array containing 609,658 SNPs uniformly distributed over the 16 *J. regia* chromosomes (Marrano, Martínez‐García et al., 2019). These SNPs were then filtered through several criteria described previously (Bernard et al., 2020), such as SNP call rate > 90%, minor allele frequency > 5%, and redundancy in the genome (SNP probes aligning in duplicated regions). Finally, 364,275 polymorphic SNPs were retained for GPs.

### 2.4 Genomic predictions

**FIGURE 1:**
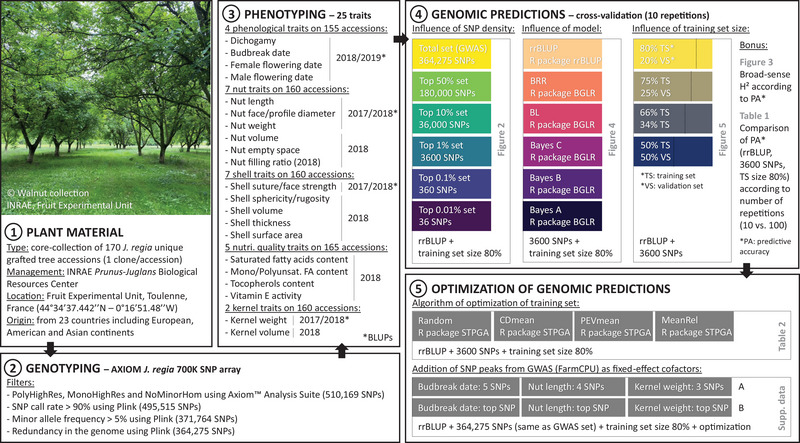
Pipeline of the analyses, including detailed information about plant material, genotyping, phenotyping, genomic predictions, and optimization. BGLR, Bayesian generalized linear regression; GWAS, genome‐wide association study; INRAE, Institut National de Recherche pour l'Agriculture, l'Alimentation et l'Environnement; PEV, percentage of explained variance; SNP, single nucleotide polymorphism; STPGA, selection of training populations with a genetic algorithm; rrBLUP, ridge regression best linear unbiased prediction.

A global overview of the pipeline of the analyses conducted in this study is given in Figure 1.

We tested six statistical models for GPs, including rrBLUP implemented in the R package “rrBLUP” (Endelman, 2011), and five Bayesian models implemented in the R package “BGLR” (Pérez & de los Campos, 2014). The rrBLUP model assumes that all SNPs contribute equally to the trait, with small effects and equal variance across markers. It is a commonly used method due to its computational efficiency and robust performance, particularly for traits controlled by many small‐effect loci. Bayesian models differ from rrBLUP by allowing heterogeneous marker variances, which can better capture the genetic architecture of complex traits. These models include: (i) BayesA that assumes each SNP with the same minor allele frequency has a different variance, following a scaled‐t distribution (suitable for traits with SNP with small and large effects), (ii) BayesB that can be seen as a complement to BayesA and assumes a fraction of SNPs are not in linkage disequilibrium (LD) with no gene and must have zero effect, while the remaining have large effects with a t‐distributed variance (suitable for trait with sparse genetic architecture), (iii) BayesC that is similar to BayesB but with a Gaussian prior for nonzero SNP effects (compromise between sparsity and polygenic architecture), (iv) Bayesian LASSO (BL) that applies a double‐exponential prior to SNP effects, inducing shrinkage while allowing for larger effect sizes (suitable for moderately sparse genetic architecture), and (v) Bayesian ridge regression (BRR) that is similar to rrBLUP but with a Gaussian prior on SNP effects. Using “BGLR,” we used the following parameters: “nIter = 6000” (total number of iterations ran by the Markov Chain Monte Carlo [MCMC] algorithm) and “burnIn = 1000” (number of initial iterations to discard in order to allow the MCMC chain to reach a stationary distribution), after verifying convergence. For each trait and each model, we conducted a cross‐validation procedure repeated 10 times, using a partitioning strategy (training set and validation set). We considered Pearson correlation between predicted and observed phenotype as a measure of the prediction accuracy. For rrBLUP, 3600 SNPs, and 80% training set size, we compared the prediction accuracy (PA) according to the number of repetitions in the random cross‐validation, 10 versus 100, to verify consistency.

### 2.5 Influence of SNP density and training set size on the accuracy of genomic predictions

Because some factors influence the prediction accuracy, we tested the impact of the SNP density and the training set size. Concerning the impact of the SNP density on the PA, we tested six sets of “top” SNPs selected according to their variance (considering the marker effects matrix) using rrBLUP model and a training set size of 80%: total set (364,275 SNPs), ∼50% set (180,000 SNPs), ∼10% set (36,000 SNPs), ∼1% set (3600 SNPs), ∼0.1% set (360 SNPs), and ∼0.01% set (36 SNPs). Concerning the impact of the training set size on the PA, we tested four sizes using rrBLUP model and the set of 3600 SNPs: random training set composed of 80% of the phenotyped accessions of the core‐collection (and remaining 20% for the validation set), 75% versus 25%, 66% versus 34%, and 50% versus 50% (Figure 1).

### 2.6 Optimization of the training set and accounting for genetic architecture using SNP peaks from GWAS

Using the R package “STPGA” (Akdemir, 2017) and the most robust scenario previously identified for all traits (rrBLUP model, the set of 3600 SNPs, and a training set size of 80%), we tested the impact on the PA of four algorithms of optimization of the training set: no optimization (or random composition), CDmean (Rincent et al., 2012), PEVmean (Rincent et al., 2012), and mean relatedness (MeanRel) based on the mean genomic additive relationship between each accession of the training set and all accessions in the validation set (VanRaden, 2008). We tested as well the addition of SNP peaks from GWAS as fixed‐effect cofactors for PHENO_bd (five SNPs), NUT_l (four SNPs), and KERNEL_w (three SNPs) based on previous marker‐trait associations identified in studies mentioned (Bernard et al., 2021, 2020), using rrBLUP model, the total set of 364,275 SNPs identical to GWAS analyses, and a training set size of 80% optimized or not (Table S2; Figure 1).

## 3 RESULTS

### 3.1 Influence of SNP density on prediction accuracy using rrBLUP and a training set size of 80%

**FIGURE 2:**
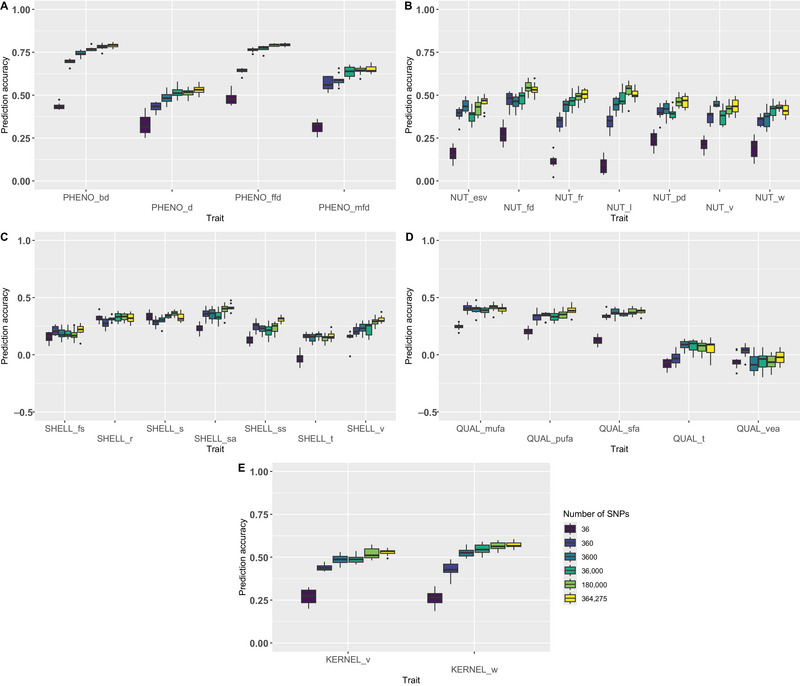
Prediction accuracies obtained using ridge regression best linear unbiased prediction (rrBLUP) and a training set size of 80% according to the single nucleotide polymorphism (SNP) density (364,275, 180,000, 36,000, 3600, 360, and 36 SNPs) for (A) phenological traits, (B) nut traits, (C) shell traits, (D) nutritional quality traits, and (E) kernel traits. General trend: traits related to phenology, nut, and kernel are well predicted, contrary to traits related to shell and nutritional quality; for most of the traits, 3600 SNPs are sufficient to give satisfying PA.

We tested six sets of SNPs selected according to their variance using rrBLUP model and a training set size of 80% to observe the impact of the SNP density on the PA: total set (364,275 SNPs), 50% set (180,000 SNPs), 10% set (36,000 SNPs), 1% set (3600 SNPs), 0.1% set (360 SNPs), and 0.01% set (36 SNPs). For all traits and all SNPs sets, the mean PA is 0.36 (Figure 2). The lowest PA (−0.20) is obtained for QUAL_vea with 36,000 SNPs, while the highest PA (0.81) is obtained for PHENO_bd with the total set. By considering the five types of traits, the best predicted are phenological traits (mean PA = 0.61), followed by kernel traits (mean PA = 0.47), nut traits (mean PA = 0.39), shell traits (mean PA = 0.25), and nutritional quality traits (mean PA = 0.20).

Generally speaking, the larger the SNP set, the better the PA obtained (Figure 2). For instance, for PHENO_bd and PHENO_ffd, the PA reaches more than 0.74 for 364,275 SNPs, 180,000 SNPs, 36,000 SNPs, and 3600 SNPs, but decreases for 360 SNPs (0.69 for PHENO_bd and 0.64 for PHENO_ffd), and decreases more for 36 SNPs (0.44 for PHENO_bd and 0.48 for PHENO_ffd). The drop in PA is all the more observable for the types of traits that are better predicted (phenology and kernel), compared to those that are less well predicted inaverage (nutritional quality). However, if both 36 and 360 SNPs sets are not sufficient to obtain satisfying PA, a plateau is obtained for most of the traits with only 3600 SNPs.

### 3.2 Correlation between broad‐sense heritability and prediction accuracy using rrBLUP, 3600 SNPs, and a training set size of 80%

**FIGURE 3:**
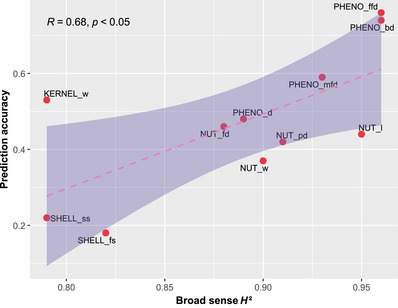
Broad‐sense heritability (*H* ^2^) values of 11 traits phenotyped over two consecutive years, depending on PA for ridge regression best linear unbiased prediction (rrBLUP), 3600 single nucleotide polymorphisms (SNPs), and a training set size of 80%. The pink dashed line represents the regression line (linear model). *R* is the Pearson correlation coefficient and *p* is the *p*‐value measuring the significance of the correlation. PA is significantly positively correlated with *H* ^2^.

For the 11 traits for which we have two consecutive years of phenotyping data, we calculated the broad‐sense heritability values (Table S1). Phenological traits are highly heritable (e.g., for PHENO_bd with *H* ^2^ = 0.96) and shell and kernel traits have a lower heritability (e.g., for KERNEL_w with *H* ^2^ = 0.79). We calculated the correlation between *H* ^2^ values and PA using rrBLUP, 3600 SNPs, and a training set size of 80% (Figure 3). With a Pearson correlation coefficient *R* = 0.68 and a *p*‐value < 0.05, *H* ^2^ is positively correlated with PA, indicating that the higher the heritability of the trait, the better the PA obtained. One exception is KERNEL_w, for which the PA is high (0.53), despite a lower *H* ^2^ (0.79).

### 3.3 Influence of prediction model on prediction accuracy using 3600 SNPs and a training set size of 80%

**FIGURE 4:**
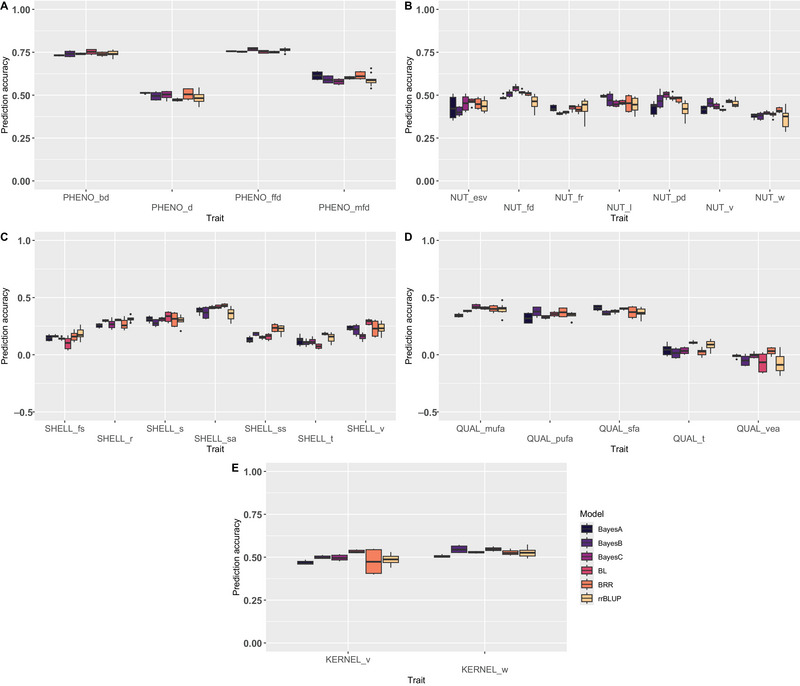
Prediction accuracies obtained using 3600 single nucleotide polymorphisms (SNPs) and a training set size of 80% according to the prediction model (BayesA, BayesB, BayesC, Bayesian LASSO [BL], Bayesian ridge regression [BRR], and ridge regression best linear unbiased prediction [rrBLUP]) for (A) phenological traits, (B) nut traits, (C) shell traits, (D) nutritional quality traits, and (E) kernel traits. General trend: the range of variation of PA between models is limited; BRR gives more unstable PA; rrBLUP performs in the average. LASSO, least absolute shrinkage and selection operator.

We tested six statistical models using 3600 SNPs and a training set size of 80% to observe the impact on the PA: BayesA, BayesB, BayesC, BL, BRR, and rrBLUP. The models perform differently according to the trait (Figure 4), but the range of variation is limited for all the traits. For the types of traits that are better predicted (phenology and kernel), the variation of PA between models is very low inaverage. For example, for PHENO_bd, the PA varies from 0.73 with BayesA to 0.75 with BL model.

However, some variations can be observed between models. For instance, BayesA gives higher PA for PHENO_d (PA = 0.51 vs. 0.49 in average for the five other models) or QUAL_sfa (PA = 0.41 vs. 0.38 in average for the five other models); however, it gives worst PA for SHELL_ss (PA = 0.13 vs. 0.19 in average for the five other models) and KERNEL_w (PA = 0.51 vs. 0.53 in average for the five other models). Using a random cross‐validation of 10 repetitions, BRR tends to give more unstable PA when observing the dispersion of the boxplots. For example, for KERNEL_v, BRR gives a PA of 0.47 ± 0.07, while the five other models give an average PA of 0.50 ± 0.03. This tendency is also observable for PHENO_d, NUT_l, SHELL_s, and QUAL_sfa, so for the five types of traits. The model rrBLUP performs as well as the other models in general: for highly heritable traits such as phenological traits or kernel traits, rrBLUP is in the average model, but for nut traits such as NUT_fd and NUT_pd, which are less heritable, rrBLUP is not necessarily the best model.

### 3.4 Influence of training set size on prediction accuracy using rrBLUP and 3600 SNPs

**FIGURE 5:**
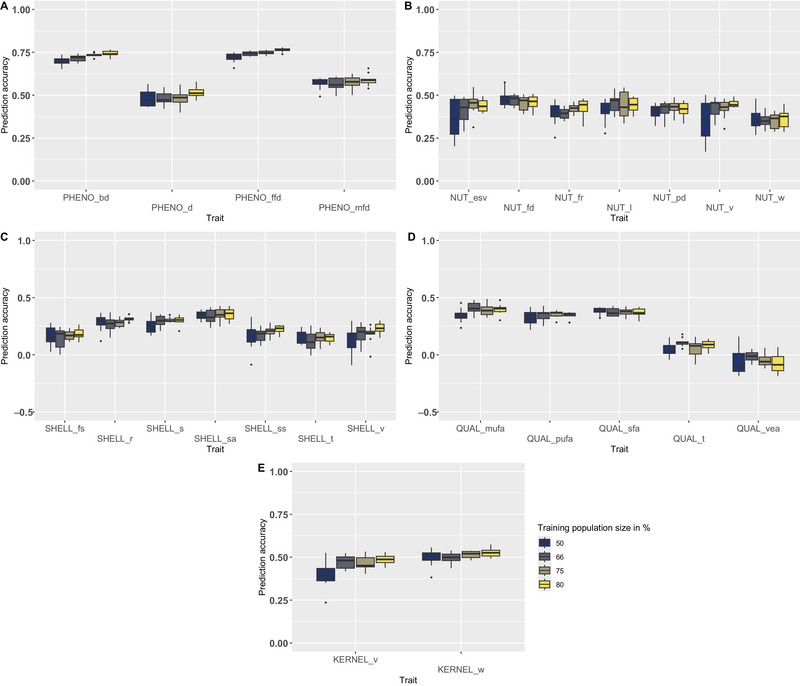
Prediction accuracies obtained using ridge regression best linear unbiased prediction (rrBLUP) and 3600 single nucleotide polymorphisms (SNPs) according to the training set size (80%, 75%, 66%, and 50%) for (A) phenological traits, (B) nut traits, (C) shell traits, (D) nutritional quality traits, and (E) kernel traits. General trend: a larger training population size gives higher PA.

We tested four training set sizes using rrBLUP model and 3600 SNPs to observe the impact on the PA: 80%, 75%, 66%, and 55%. As previously observed for the SNP density, the larger the training set size, the better the PA obtained (Figure 5). However, differences are very low (e.g., for PHENO_bd, between 0.74 using 80% and 0.70 using 50%), and the dispersion of the boxplots is higher with a training set size of 50%, as we can particularly observe for NUT_esv (PA = 0.37 ± 0.11 vs. 0.43 ± 0.06 in average for the three largest training set sizes) and NUT_v (PA = 0.35 ± 0.12 vs. 0.43 ± 0.05 in average for the three largest training set sizes), for which the range of the phenotypic data is the larger (Table S1).

We compared, for rrBLUP, a training set size of 80%, and 3600 SNPs, the random cross‐validation using 10 or 100 repetitions. Results indicate that means and standard deviations are highly similar for the 25 traits between 10 or 100 repetitions (Table 1). For example, a PA of 0.59 ± 0.03 is obtained for PHENO_mfd using 10 or 100 repetitions. Using 100 repetitions does not necessarily decrease the standard deviation (e.g., for NUT_v, PA = 0.45 ± 0.02 using 10 repetitions vs. 0.40 ± 0.06 using 100 repetitions).

**TABLE 1:** Comparison of prediction accuracy for rrBLUP, 3600 SNPs, and 80% training set size according to the number of repetitions in the random cross‐validation.

Trait	Mean ± standard variation for 10 repetitions	Mean ± SD for 100 repetitions
PHENO_d	0.48 ± 0.03	0.49 ± 0.03
PHENO_bd	0.74 ± 0.02	0.73 ± 0.02
PHENO_ffd	0.76 ± 0.01	0.76 ± 0.01
PHENO_mfd	0.59 ± 0.03	0.59 ± 0.03
NUT_l	0.44 ± 0.04	0.46 ± 0.05
NUT_fd	0.46 ± 0.04	0.48 ± 0.04
NUT_pd	0.42 ± 0.04	0.43 ± 0.04
NUT_v	0.45 ± 0.02	0.40 ± 0.06
NUT_esv	0.44 ± 0.03	0.43 ± 0.04
NUT_fr	0.43 ± 0.05	0.42 ± 0.04
NUT_w	0.37 ± 0.06	0.35 ± 0.05
SHELL_s	0.30 ± 0.04	0.29 ± 0.04
SHELL_r	0.31 ± 0.03	0.29 ± 0.04
SHELL_v	0.23 ± 0.05	0.21 ± 0.06
SHELL_t	0.15 ± 0.04	0.14 ± 0.04
SHELL_sa	0.36 ± 0.05	0.38 ± 0.05
SHELL_ss	0.22 ± 0.03	0.21 ± 0.04
SHELL_fs	0.18 ± 0.05	0.16 ± 0.05
QUAL_sfa	0.37 ± 0.04	0.39 ± 0.03
QUAL_mufa	0.39 ± 0.05	0.41 ± 0.03
QUAL_pufa	0.34 ± 0.03	0.36 ± 0.04
QUAL_t	0.08 ± 0.04	0.06 ± 0.04
QUAL_vea	−0.08 ± 0.08	−0.05 ± 0.06
KERNEL_v	0.49 ± 0.03	0.48 ± 0.04
KERNEL_w	0.53 ± 0.02	0.51 ± 0.03

Abbreviations: rrBLUP, ridge regression best linear unbiased prediction; SNP, single nucleotide polymorphism.

### 3.5 Optimization of the training set using CDmean, PEVmean, and MeanRel algorithms

We tested four algorithms of optimization for a training set size of 80% using rrBLUP model and 3600 SNPs to observe the impact on the PA: random, CDmean, PEVmean, and MeanRel criteria. Results show that PA is higher in most cases (20 out of 25 traits) when an optimization algorithm is applied (Table 2). CDmean, PEVmean, and MeanRel give highest PA for five, seven, and nine traits, respectively. When considering the type of traits, MeanRel gives lowest PA for most of phenological traits, but highest PA for most of shell traits. For instance, for PHENO_mfd, MeanRel gives a PA of 0.22, whereas CDmean, PEVmean, and random give a PA higher than 0.60; however, for SHELL_ss, all optimization methods give a PA lower than 0.29, except for MeanRel for which PA = 0.54. Within a type of trait, the use of CDmean or PEVmean leads to contrasted results according to the trait. As an example, when considering the nut traits, CDmean and PEVmean give higher PA for NUT_l and NUT_fr but lower PA for NUT_pd, NUT_v, and NUT_w. This observation is the same when considering shell traits, for which CDmean and PEVmean give higher PA for SHELL_s, SHELL_t, and SHELL_sa, but lower for SHELL_v, SHELL_ss, and SHELL_fs. We calculated the gain of each optimization method for all traits (Table 2). Considering all traits, the average gains using CDmean, PEVmean, and MeanRel are 24%, 37%, and 8%, respectively, compared to random. The results confirm the best PA for shell traits using MeanRel (e.g., a gain of 104% for SHELL_s), with an average gain of 60% compared to random.

**TABLE 2:** prediction accuracies obtained using rrBLUP and 3600 SNPs according to the training set optimization algorithm for a training set size of 80%.

Trait	Random Mean ± SD	CDmean Mean ± SD	PEVmean Mean ± SD	MeanRel Mean ± SD	Gain % CDmean	Gain % PEVmean	Gain % MeanRel
PHENO_d	0.49 ± 0.15	0.44 ± 0.06	0.45 ± 0.08	0.47 ± 0.00	−10	−8	−4
PHENO_bd	0.75 ± 0.06	0.80 ± 0.01	0.80 ± 0.01	0.55 ± 0.00	7	7	−27
PHENO_ffd	0.77 ± 0.08	0.78 ± 0.01	0.79 ± 0.01	0.66 ± 0.00	1	3	−14
PHENO_mfd	0.61 ± 0.11	0.65 ± 0.03	0.63 ± 0.02	0.22 ± 0.00	7	3	−64
NUT_l	0.47 ± 0.13	0.54 ± 0.01	0.51 ± 0.01	0.56 ± 0.00	15	9	19
NUT_fd	0.46 ± 0.08	0.49 ± 0.09	0.44 ± 0.06	0.50 ± 0.00	7	−4	9
NUT_pd	0.50 ± 0.09	0.46 ± 0.04	0.39 ± 0.05	0.30 ± 0.00	−8	−22	−40
NUT_v	0.46 ± 0.20	0.46 ± 0.01	0.39 ± 0.07	0.43 ± 0.00	0	−15	−7
NUT_esv	0.46 ± 0.11	0.52 ± 0.02	0.43 ± 0.05	0.50 ± 0.00	13	−7	9
NUT_fr	0.48 ± 0.10	0.53 ± 0.00	0.56 ± 0.05	0.44 ± 0.00	10	17	−8
NUT_w	0.35 ± 0.25	0.27 ± 0.08	0.20 ± 0.07	0.28 ± 0.00	−23	−43	−0
SHELL_s	0.23 ± 0.16	0.26 ± 0.02	0.29 ± 0.01	0.47 ± 0.00	13	26	104
SHELL_r	0.30 ± 0.17	0.25 ± 0.02	0.29 ± 0.01	0.47 ± 0.00	−17	−3	57
SHELL_v	0.23 ± 0.20	0.15 ± 0.07	0.12 ± 0.09	0.27 ± 0.00	−35	−48	17
SHELL_t	0.11 ± 0.12	0.20 ± 0.10	0.21 ± 0.15	0.17 ± 0.00	82	91	55
SHELL_sa	0.35 ± 0.11	0.40 ± 0.00	0.32 ± 0.07	0.42 ± 0.00	14	−9	20
SHELL_ss	0.28 ± 0.15	0.16 ± 0.03	0.02 ± 0.14	0.54 ± 0.00	−43	−93	93
SHELL_fs	0.14 ± 0.10	0.08 ± 0.03	0.09 ± 0.10	0.24 ± 0.00	−43	−36	71
QUAL_sfa	0.45 ± 0.11	0.46 ± 0.02	0.47 ± 0.04	0.37 ± 0.00	2	4	−18
QUAL_mufa	0.38 ± 0.13	0.49 ± 0.03	0.48 ± 0.01	0.18 ± 0.00	29	26	−53
QUAL_pufa	0.38 ± 0.09	0.33 ± 0.01	0.35 ± 0.03	0.13 ± 0.00	−13	−8	−66
QUAL_t	0.11 ± 0.12	0.24 ± 0.10	0.27 ± 0.04	0.20 ± 0.00	118	145	82
QUAL_vea	0.02 ± 0.09	0.12 ± 0.11	0.21 ± 0.03	0.02 ± 0.00	500	950	0
KERNEL_v	0.49 ± 0.14	0.46 ± 0.05	0.41 ± 0.04	0.43 ± 0.00	−6	−16	−12
KERNEL_w	0.47 ± 0.07	0.39 ± 0.08	0.30 ± 0.04	0.47 ± 0.00	−17	−36	0
				Mean	24	37	8

*Note*: Best prediction accuracies considering standard deviation are given in gray. Gain in prediction accuracy is estimated as follows: [(Optimum method − Random)/Random] × 100.

Abbreviations: CDmean, coefficient of determination mean; MeanRel, mean relatedness; PEVmean; prediction error variance mean; rrBLUP, ridge regression best linear unbiased prediction; SNP, single nucleotide polymorphism.

We compared, for rrBLUP, a training set size of 80%, the 0.1% set of 3600 SNPs, and the random cross‐validation using 10 repetitions, the PA given by using the “rrBLUP” R package or using the “STPGA” R package, the latter automatically implementing a step of dimensionality reduction of the genotyping matrix using the singular value decomposition (SVD) method. Results indicate that means are highly similar for the 25 traits (Table S3). For example, a PA of 0.74 ± 0.02 is obtained for PHENO_bd not considering SVD, or PA = 0.75 ± 0.06 considering SVD. However, the standard deviation is higher considering SVD (e.g., for NUT_w, PA of 0.37 ± 0.06 not considering SVD and 0.35 ± 0.25 considering SVD).

### 3.6 Addition of SNP peaks from GWAS as fixed‐effect cofactors

For three traits (PHENO_bd, NUT_l, and KERNEL_w), we added SNP peaks from GWAS previously conducted, as fixed‐effect cofactors using rrBLUP model, the same set of 364,275 SNPs as used in GWAS, and a training set size of 80%, to observe the impact on the PA. We tested two configurations: including all significant SNPs and only the “top” SNP based on significance. For PHENO_bd, five SNPs were previously identified including two with a percentage of explained variance (PEV) higher than 25% using FarmCPU model (Table S2). For NUT_l and KERNEL_w, four and three SNPs were previously identified, respectively, with a PEV lesser than 15% using Fixed and random model Circulating Probability Unification (FarmCPU) model.

Regarding PHENO_bd, the addition of the five SNPs associated to the trait by GWAS as fixed‐effect cofactors drastically decreases the PA. Without optimization, a PA of 0.78 is obtained, and when SNPs are included as fixed‐effect cofactors, the PA drops to 0.39 (Table S4). The PA is drastically decreased as well when applying a training set optimization algorithm such as CDmean or PEVmean (e.g., for PHENO_bd and CDmean: PA = 0.78 vs. when SNPs are included as fixed‐effect cofactors: PA = 0.56). For NUT_l, the results are similar, with an average PA of 0.25 and 0.46, when including or not the four SNPs associated to the trait by GWAS as fixed‐effect cofactors, respectively. Concerning KERNEL_w, the combination of training set optimization algorithm and addition of three SNPs peaks from GWAS as fixed‐effect cofactors can slightly increase the prediction (e.g., for PEVmean, PA of 0.34, and when SNPs are included as fixed‐effect cofactors, PA of 0.35).

When including only the “top” SNP identified previously in GWAS as one single fixed‐effect cofactor, the decrease of PA is much more limited. For instance, for PHENO_bd, we previously observed a decrease of PA of 0.39 in random condition including the five significant SNPs, but the decrease is only 0.05 in random condition as well including only the “top” SNP. We still observe a slight increase of PA for KERNEL_w when using the combination of CDmean or PEVmean and “top” SNP as fixed‐effect cofactor (e.g., PA = 0.33 with PEVmean and PA = 0.36 with PEVmean + “top” SNP).

## 4 DISCUSSION

The objective of this study was to explore, for the first time, the potential of GP in walnut, regarding traits related to phenology and walnut quality, using a core‐collection of 170 accessions. We investigated three factors influencing PA: the SNP density (from total set of the Axiom *J. regia* 700K SNP used for GWAS to 0.01% set of 36 SNPs), the model used (rrBLUP, Bayesian alphabet, BL, and BRR), and the training set size (from 80% to 50%). We explored as well two optimization levers: the optimization of the composition of the training set (using CDmean, PEVmean, and MeanRel algorithms), and accounting for prior information about genetic architecture of the traits, based on GWAS (using SNPs associated to the trait as fixed‐effect cofactors).

### 4.1 Genomic prediction in walnut using a core‐collection and the Axiom *J. regia* 700K SNP is promising

Walnut breeders are compelled to wait 6–7 years before observing the first fruit characteristics of any hybrid resulting from a biparental cross. Our study revealed that GP within a diversity panel gives promising results in walnut, since PA reached 0.74 for PHENO_bd and 0.53 for KERNEL_w (rrBLUP, 3600 SNPs, training size of 80%), two major traits related to phenology and fruit quality, respectively. Because of the long generation time and juvenile period, such promising results can give new perspectives in any new walnut breeding program. In diversity panels, typically consisting of unrelated individuals, a broader allelic diversity of the species is represented. This leads generally to a faster LD decay along the genome, contrary to family‐based panels, in which large blocks with high LD come from shared ancestry. A broad genetic background could make harder the evaluation of the effect of SNPs on the traits in diversity panel, potentially leading to lower PA for specific traits, but may make it more applicable across diverse populations. In other words, high PA obtained within a family can drop dramatically when the trained model is applied to a progeny from unrelated populations. Ben Hassen et al. (2018) showed in rice that maximum PA of 0.65, obtained with a model trained within a diversity panel of 284 accessions for “days to flowering” trait, drops to 0.51 in 97 inbred lines from 36 biparental crosses, involving 31 accessions of the diversity panel. This study confirms this statement, but also the reliability of such diversity panels to predict progenies, especially if they come from parents included in the diversity panel.

In annual species, such as pea, a diversity panel of 372 accessions was investigated as well to predict complex traits, such as “beginning of flowering,” for which PA varied from 0.28, using SPLS‐DAPC model, to 0.46 using genomic best linear unbiased prediction (GBLUP) model, for the same year of phenotyping (Burstin et al., 2015). However, on perennial species, many investigations of GP involved an F_1_ pseudo‐testcross population as training set, as conducted in apricot (Nsibi et al., 2020), or several families, as conducted in citrus (Minamikawa et al., 2017) and pear (Kumar et al., 2019). Fortunately, as we have done on walnut, several studies investigated the reliability of GP models trained using a diversity panel, particularly in grapevine. A diversity panel of 279 grapevine accessions was genotyped with SNPs from genotyping‐by‐sequencing and microarray, and both datasets are suitable to train an rrBLUP model to predict 152 response variables, with an average PA of 0.42 (Flutre et al., 2022). Brault et al. (2022) trained models from the same diversity panel and 15 traits including phenology and fruit quality, and tested 10 pseudo‐F_1_ biparental families obtained by crossing five cultivars: an average per‐trait PA of 0.60 was obtained at the scale of the cross mean. In apple, Roth et al. (2020) investigated the GP of fruit texture using acoustic and mechanical measurements in biparental families using a training set consisting of 259 accessions and PA varied depending on the family considered.

When comparing these findings to walnut, our study supports the trend observed in other fruit species that diversity panels can be used effectively for GP. Despite having only 170 accessions, the INRAE *Prunus*‐*Juglans* Biological Resources Center core collection could still serve as a valuable GP resource. This suggests that our core collection is well‐defined and suitable for key traits such as phenology, nut size, and kernel size, helping to reduce both phenotyping and genotyping costs and time.

### 4.2 Several factors influence PA and choice of the model has to be made according to the trait

Several factors can influence PA including the heritability of the trait as it was shown in previous studies (Desta & Ortiz, 2014; Poland et al., 2012). We observed in walnut as well a positive correlation between PA and broad‐sense heritability. Since phenological traits are highly heritable, it is not surprising to obtain high PA. However, KERNEL_w is surprisingly well predicted while less heritable compared to SHELL_ss for which *H* ^2^ is the same (0.79). This result cannot be explained by the range of the BLUPs. In fact, we investigated the level of correlation between the range of the phenotypic data of the 25 traits and PA for rrBLUP, 3600 SNPs, and a training set size of 80% (data not shown), and results showed no correlation (Pearson correlation coefficient *R* = 0.09 and a *p*‐value = 0.7). This indicates that PA is not influenced by the level of variability of the trait, which is, however, extremely different in our study: We have a low range considering the BLUPs values for the traits phenotyped over 2 years, but a high range for the other traits phenotyped only 1 year. The high PA of KERNEL_w may be due to hypothetical high LD between SNPs and causal variants that may enhance the model's ability to capture the genetic architecture of the trait.

A previous study on pepper highlighted that highly correlated traits showed similar patterns of PA between the models tested (Hong et al., 2020). We observed this tendency as well in walnut. For phenological traits, PHENO_bd and PHENO_ffd are highly positively correlated with a coefficient of correlation of 0.83 (Bernard et al., 2020), and both traits share the same pattern: a PA of 0.74 and 0.76, respectively, with no important difference between models. For nut traits, NUT_fd and NUT_pd, positively correlated with *r* = 0.87 (Bernard et al., 2021), the pattern was similar as well: the best PA was obtained with BayesC (0.54 and 0.50, respectively), and the worst PA was obtained with rrBLUP (0.46 and 0.42, respectively).

Not surprisingly, a higher density of SNPs led to a better PA, thanks to a higher probability to include markers in LD with the causal variants. This was already demonstrated in apricot (Nsibi et al., 2020), in which a plateau is reached with 6103 SNPs, representing only 10% of the total set of markers, which is comparable to our study, in which a plateau is tended to be reached with 3600 SNPs. While increasing SNP density generally improves PA, the gain is not linear and adding more SNPs may bring marginal improvements, particularly if they are in strong LD with existing ones. Increasing SNP density can even sometimes reduce the PA, such as for SHELL_s in our study, probably because of multicollinearity between markers, that leads to overfitting the model (Muir, 2007). For practical purposes, identifying the optimal minimum number of SNPs needed to achieve sufficient PA while reducing computational requirements, is fundamental for developing a new low‐density array that would be cost‐effective and useful in breeding program.

The method of SNP density reduction can have an impact as well. In our total set of SNPs, a previous filtering based on minor allele frequency and redundancy in the genome was conducted for GWAS analyses. Then, to select the reduced set (top 50%, top 10%, etc.), a choice was made based on the variance of the SNPs, that is to say on their level of polymorphism. However, such method may lead to a bias if some genomic regions are more polymorphic, and another solution would be to reduce the SNP density based on LD pruning, in order to keep all the potential genomic regions associated to a trait. In our study, similarly to a higher SNP density, a higher training set size led to a better PA, as it was observed as well in apricot (Nsibi et al., 2020), for which a training set size of 75% systematically gives a higher PA for all traits compared to a size of 50%.

For most of our traits studied, the impact of the model on PA was observable, but not tremendous, compared to the work of Heslot et al. (2012). There was no model that clearly outperforms the others. For instance, for the four phenological traits, the best model was different (BL, BRR, BayesA, or BayesC depending on the trait). Regarding the relatively fast computational time of rrBLUP model (Ahmadi et al., 2021), one can consider to test this model first in order to obtain an idea of the ability to predict one trait. However, a limitation in using rrBLUP would be assumption of equal contribution of all SNPs to the variation of the trait, that may not be suitable for oligogenic traits governed by major loci. In the case of a trait with few underlying loci of large effect, Bayesian models will outperform rrBLUP, as it was demonstrated in loblolly pine (Resende et al., 2012). The choice of model depends on the trait and whatever the model, some traits are not well predicted. In our study, this is the case for two traits, QUAL_t and QUAL_vea, that even reached negative PA, indicating that the prediction models underperformed than a random permutation. Tocopherols are vitamin E derivatives with antioxidant properties, and together with vitamin E activity, they are both traits that were quantified in one single year. A work conducted by Amaral et al. (2005) showed that, besides significant differences in tocopherols content between nine walnut cultivars, these differences were significant as well considering 3 years of walnut production, indicating that vitamin E composition is influenced by environmental factors. Even though walnut is a valuable source of vitamin E, considering that 100 g of kernels provides 140% of daily need (Şen & Karadeniz, 2015), the content may be more dependent on orchard management and climatic conditions than genotype, potentially leading to a low heritability of this trait.

### 4.3 Optimization levers are still to be investigated

Our results highlighted the contribution of CDmean, PEVmean, and MeanRel as training set optimization methods for some traits using rrBLUP, but not systematically. Our results were not able to give a trend. For instance, PEVmean was the best method for seven traits but the worst for 10 traits. Similarly, using MeanRel gave the best PA for nine traits, but the worst for nine other traits, while using CDmean gave the best and worst PA for five and three traits, respectively. CDmean takes into account covariance among genotypes and prevents the selection of closely related individuals (Isidro y Sánchez et al., 2015; Rincent et al., 2012). While this can be beneficial for maximizing genetic diversity, it may introduce higher variability in cross‐validation, reducing PA stability. In our study, CDmean was a stable method, considering the standard deviation, but did not emerge as a universally effective method for all our traits. PEVmean was the best method for nine traits in walnut and has been shown to outperform random method in winter wheat (Sarinelli et al., 2019). However, it also performed poorly for other traits, suggesting that its benefits are trait‐dependent. MeanRel, which prioritizes genetic relatedness, consistently selects highly related individuals. This reduces variability but may also lead to overfitting and redundancy in cross‐validation.

In our study, the average gain across all traits was positive for the three optimization methods. However, high levels of gain were observed for QUAL_t and QUAL_vea using CDmean and PEVmean because the PA obtained without optimization was tremendously low. Although the gain seemed substantial, the PA after optimization remained low. When discarding these two traits to estimate the gain, the average gains were −1%, −7%, and 8% for CDmean, PEVmean, and MeanRel, respectively. The higher potential gain of MeanRel was due to its superior capacity to predict shell‐related traits only. This suggests that MeanRel may be more suitable for traits influenced by highly related genetic backgrounds, while CDmean and PEVmean may be more beneficial when maximizing genetic diversity is important. The challenge lies in determining which traits benefit more from selecting related versus distant individuals.

In our core‐collection of 170 accessions, a structure of *K* = 2 was previously determined, and no specific effect of this structure was shown on the traits in average (Bernard et al., 2021, 2020). It means that, by using principal component analysis, there is no group that contains more large‐fruited accessions or earlier accessions in phenology. However, it would be interesting to verify, trait by trait, the proportion of representativeness of each group according to the optimization method. Our results tended to show that the method of training set optimization has more impact than the choice of the model on PA, and this was also proved by Fernández‐González et al. (2023).

Nsibi et al. (2020) in apricot showed that the inclusion of GWAS markers as fixed effect cofactors can improve PA using rrBLUP for most of fruit quality traits tested. However, adding SNPs from GWAS as cofactors worsened most of PA in our study, even though the same panel, the same phenotypic data and the same genotypic data were used for both GWAS and GP. We considered SNP peaks from GWAS previously identified using FarmCPU (Bernard et al., 2021, 2020), and while this model can reduce confounding effects, it does not model epistatic interactions (X. Liu et al., 2016); if the GWAS SNPs are tightly linked, including them as fixed effects can lead to overfitting in the GP model. We did not investigate epistatic interactions for our traits in walnut but flowering date, for instance, is a trait known to involve epistasis in barley (Afsharyan et al., 2020), soybean (K. H. Kim et al., 2020), and rice (C. Liu et al., 2021). Epistatic interactions may play a role as well in fruit tree species flowering, as it was already observed in apricot (Morgane Roth, personal communication, April 2, 2024). Bayesian methods can better accommodate the inclusion of SNPs from GWAS by assigning different priors to the SNP effects, contrary to methods based on regression such as rrBLUP. When comparing the results by the inclusion of all significant SNPs or only the “top” SNP, we clearly observed a lower decrease of PA, indicating a lower overfitting of the model. However, we would not suggest to include SNP from GWAS as fixed‐effect cofactors to improve PA in our experimental design.

### 4.4 Comparison of GWAS and GP based on performance metrics

In order to know if GS is a promising method for walnut breeding compared to more classical MAS based on GWAS results, we compiled for each trait a table summarizing key statistics (Table 3). We chose the following criteria to evaluate the performance of both methods: (i) MAS is suggested if less than three SNPs explain a percentage of variance higher than 30% (PEV > 30%) based on GWAS results using FarmCPU model previously identified (Bernard et al., 2021, 2020); (ii) GS is suggested if PA is higher than 0.40.

**TABLE 3:** Comparison of performance metrics between GWAS using FarmCPU and GP using rrBLUP for suggesting a selection method (marker‐assisted selection [MAS] or genomic selection [GS]).

Trait	GWAS significant SNPs	Top SNP PEV (%)	Cumulative top three SNP PEV (%)	Mean GP accuracy	Suggested method^a^
PHENO_d	1	77.2	77.2	0.48	MAS/GS
PHENO_bd	5	30.6	64.7	0.74	MAS/GS
PHENO_ffd	2	36.2	67.4	0.76	MAS/GS
PHENO_mfd	2	6.1	8.0	0.59	GS
NUT_l	4	15.4	44.4	0.44	MAS/GS
NUT_fd	4	26.4	52.0	0.46	MAS/GS
NUT_pd	2	16.5	16.6	0.42	GS
NUT_v	6	17.33	50.8	0.45	MAS/GS
NUT_esv	4	17.1	50.1	0.44	MAS/GS
NUT_fr	4	20.4	42.6	0.43	MAS/GS
NUT_w	5	17.1	24.8	0.37	No method
SHELL_s	0	–	–	0.30	No method
SHELL_r	0	–	–	0.31	No method
SHELL_v	0	–	–	0.23	No method
SHELL_t	1	13.7	13.7	0.15	No method
SHELL_sa	2	16.5	32.6	0.36	MAS
SHELL_ss	2	14.8	27.0	0.22	No method
SHELL_fs	2	16.1	27.4	0.18	No method
QUAL_sfa	0	–	–	0.37	No method
QUAL_mufa	5	17.7	45.6	0.39	MAS
QUAL_pufa	0	–	–	0.34	No method
QUAL_t	8	18.2	44.8	0.08	MAS
QUAL_vea	2	13.8	19.1	−0.08	No method
KERNEL_v	3	25.0	65.5	0.49	MAS/GS
KERNEL_w	3	11.5	26.7	0.53	GS

*Note*: MAS if < 3 SNPs explain a PEV > 30%; GS if GP accuracy > 0.4.

Abbreviations: GP, genomic prediction; GWAS, genome‐wide association study; PEV, percentage of explained variance; rrBLUP, ridge regression best linear unbiased prediction; SNP, single nucleotide polymorphism.

^a^ Threshold for decision.

Based on our criteria, GS is the only method of selection suggested for 3 traits: PHENO_mfd, NUT_pd, and KERNEL_w. On the contrary, MAS is the only method of selection suggested for 3 traits: SHELL_sa, QUAL_mufa, and QUAL_t. Interestingly, both MAS and GS can be suitable for most of traits related to phenology and nut, and for KERNEL_v (Table 3). For most of shell traits and nutritional quality traits, neither method can be recommended because of missing marker‐trait association explaining a sufficient percentage of variance identified in GWAS and low PA in GP. These traits were phenotyped for one single year and it could be the reason why we did not find any associated SNP in GWAS neither satisfying PA in GP. For most of phenological traits, both methods are suitable, but given the cumulative PEV of the “top 3” SNPs identified in GWAS, we could preconize MAS as a first approach. Moreover, for PHENO_bd, a marker based on the top SNP detected in GWAS on chromosome 1 is already developed for breeding and validated using plant material from 96 breeding line accessions of the Walnut Improvement Program of the University of California, Davis (Bernard et al., 2020).

While both approaches offer valuable insights, they serve different purposes in breeding programs. GWAS is a powerful tool for identifying loci associated with key traits, facilitating MAS when major QTLs are involved. However, it is less effective for highly polygenic traits. GP, in contrast, leverages genome‐wide marker data to estimate breeding values, making it particularly useful for complex traits. In walnut breeding applications, both methods can be complementary, depending on the objectives.

### 4.5 Comparison of genomic prediction across fruit tree species and implication for walnut breeding

The application of GP has emerged as a powerful tool for accelerating breeding programs in various fruit tree species, including apple, pear, peach, sweet cherry, citrus, and grapevine. Similar to our findings in walnut, these studies consistently demonstrate the potential of GP for complex traits, particularly those related to fruit quality, often a priority in breeding (Iwata et al., 2016). In apple, more than 1000 seedlings from a factorial mating design involving four female and two male parents were genotyped using an 8K SNPs array, and rrBLUP was then applied to predict various fruit quality traits with high accuracy, such as astringency (PA = 0.67), firmness (PA = 0.83), and soluble solids content (PA = 0.89) (Kumar et al., 2012). More recently, Minamikawa et al. (2024) demonstrated that GP accuracy for traits such as skin coloration, harvest time, and malic acid content was improved by combining SNP array data with newer genotyping technologies, such as random amplicon sequencing‐direct systems (more than 22K SNPs in total), when using parental and breeding populations. In pear, a biparental population of 310 individuals was genotyped using genotyping‐by‐sequencing, and a fivefold cross‐validation with rrBLUP revealed that PA ranged from 0.33 for fruit core vertical diameter to 0.65 for stone cell content (Sun et al., 2024). Additionally, the authors showed that as few as 2000 SNPs were sufficient to achieve reasonably accurate PA.

In peach, a European study analyzed 1147 individuals from biparental populations derived from four crosses in Italy, two in France, and five in Spain. These individuals were genotyped using a 9K SNPs array (Biscarini et al., 2017). By applying the GBLUP model in a fivefold cross‐validation, PA varied considerably across crosses. However, when averaged, PA ranged from 0.72 for sugar content to 0.65 for titratable acidity and 0.60 for fruit weight. Hardner et al. (2022) further explored the integration of genotype by environment (G × E) interactions in GP using both univariate and multivariate linear models for soluble solids content, and 577 accessions from three peach breeding programs across four trial locations in the United States. The results showed that in some cases, PA was higher when data from multiple trials were combined. In grapevine, using genotyping‐by‐sequencing and individuals of two breeding programs, application of the GBLUP model in a fivefold cross‐validation revealed that PA varied from 0.50 to 0.86 across traits (Brault et al., 2024). In this study, traits related to anthocyanin content were better predicted than those related to sugar and acid content. In sweet cherry, using the GBLUP model in a fivefold cross‐validation as well, Munyengwa et al. (2023) showed that GP based on both individual SNPs and genealogy‐based haplotypes fitted as pseudo‐biallelic loci can lead to high PA for firmness (0.67). However, using pseudo‐biallelic loci instead of individual SNPs did not improve or reduce PA. As a final example, a study on citrus focusing on fruit weight, sugar content, and acid content showed that the use of single‐step GBLUP, a GP method combining the kinship information from both genotyped and non‐genotyped relatives, can improve PA for non‐genotyped individuals (Imai et al., 2019).

This review of the literature highlights many similarities across fruit tree species with a long juvenile period: the prediction of polygenic traits, the use of rrBLUP or GBLUP models, a genotyping primarily based on SNP arrays, the application of fivefold cross‐validation, and the assessment of factors such as SNP density and training population size that can influence PA. While the general methodology and objectives of predicting complex traits are similar across species, there are notable differences and unique challenges. A key factor in building robust GP models is the availability of genomic resources and appropriate plant material for training. In most fruit tree species, GP models have been trained using classical SNP data, but in walnut, complementary genotyping information such as haplotypes or sequence data is still lacking, while it is beginning to emerge in other species. Similarly, the opportunity to test models within a breeding population remains a milestone for validating trained models—yet this step has not been achieved in walnut so far. Another important aspect is the presence of species‐specific traits that probably have distinct genetic architectures. For example, the fatty acids composition is specific to nut species. As a result, there is likely less information available in the literature to guide the choice of appropriate models for these traits. Moreover, the integration of prior knowledge from quantitative genetics studies is not consistently effective. Finally, we anticipate the development of more complex models incorporating epistasis. In our study, we explored kinship matrices based on additive, dominance, and epistatic effects. The results (data not shown) suggest that allelic interactions, both within and between loci, are very limited in our panel of accessions and with the SNPs used from the array, probably because of a limited heterozygosity rate of 0.3.

Despite these challenges, GS holds significant promise for walnut breeding, as our findings align with the broader trend of applying GS to accelerate improvement in perennial fruit crops. However, the unique biological characteristics of walnut may require adapted strategies. Continued progress in developing genomic resources, refining phenotyping methods, and optimizing statistical models will be essential to fully harness the potential of GS for enhancing cultivar development in this species.

## 5 CONCLUSION

This study highlights that the use of the INRAE core‐collection of 170 walnut accessions as a training set and the Axiom *J. regia* array can give high PA for several complex traits, particularly for phenological traits that are crucial for future climate change adaptation. The results showed (i) a plateau of PA considering an SNP set of 1% from the total set of the array for all traits, (ii) a limited impact of the model and the training set size on PA for all traits, (iii) an improvement of PA when applying an optimization method for most of the traits, but (iv) an overcorrection when including SNP peaks from GWAS as fixed‐effect cofactors. For any walnut breeding program, this work can represent a valuable tool to improve the traits governed by many loci with low effects in order to reduce the time of breeding and reduce the labor in phenotyping the walnut quality of hybrids.

As a perspective, it would be interesting to test the models trained using this diversity panel, having a wide range of alleles, in an F_1_ population from a biparental cross. However, since the walnut breeding program has ceased in France, we can only hope that walnut breeders worldwide will use our data and genotype their populations using the same array, with likely better results if the parents are part of our core collection. Considering the priorities in selection—phenology and kernel—and our comparison of performance between MAS and GS, we suggest to implement MAS for selecting individuals for budbreak date, but we recommend to develop GS for selecting individuals for other traits, including the kernel weight. However, GS is more complex to implement, and breeders must be proficient in R scripts and interpreting GP results, which are often more intricate than classical MAS. Therefore, the choice of approach will ultimately depend on the breeder's financial resources and the support they can access for the analyses.

We could consider to test multi‐trait models and investigation of G × E interactions as well in a next step. Multi‐trait GS can improve PA by leveraging genetic correlations, enabling the selection of low‐heritability traits using information from correlated, higher heritability traits. However, challenges include increased model complexity, the need for large high‐quality datasets, and potential conflicts between traits with unfavorable correlations. G × E interactions can provide insights into the effects of loci associated with specific climatic conditions, but this requires planting the core collection across multiple sites with contrasting climates.

## AUTHOR CONTRIBUTIONS

**Anthony Bernard**: Conceptualization; data curation; formal analysis; visualization; writing—original draft. **Juliette Bénéjam**: Formal analysis; methodology; validation; writing—review and editing. **Morgane Roth**: Formal analysis; methodology; validation; writing—review and editing. **Fabrice Lheureux**: Funding acquisition; writing—review and editing. **Elisabeth Dirlewanger**: Funding acquisition; writing—review and editing.

## CONFLICT OF INTEREST STATEMENT

The authors declare no conflicts of interest.

## Supporting information




**Table S1**. Summary of phenotyping data available and broad‐sense heritabilities.

**Table S2**. SNPs identified associated to the traits by GWAS included as fixed‐effect cofactors (rrBLUP, 364,275 SNPs, and training set size 80%).

**Table S3**. Comparison of prediction accuracy for rrBLUP, 3600 SNPs, 80% training set size according to the consideration or not of the SVD.

**Table S4**. prediction accuracies obtained using rrBLUP, 364,275 SNPs, and a training size of 80% according to the training set optimization algorithm and the addition of SNP peaks from GWAS as fixed‐effect cofactors.

## Data Availability

The SNP genotyping raw dataset in “hapmap” format is freely and openly accessed on the “Recherche Data Gouv” French official institute repository via the identifier “INRAE's Walnut Genotyping Resources” and at: https://doi.org/10.15454/XPKII8. The dataset is called “3_GWAS_SNP_hapmap.txt.” The file called “5_List_of_ID_SNP.tab” allows to link the array identifier name of the accessions used in this study with the identifier name of the INRAE walnut germplasm collection. The phenotypic raw dataset in “csv” format is freely and openly accessed on the “Recherche Data Gouv” French official institute repository via the identifier “INRAE's Walnut Genetic Resources Observation (Experimental data 2017–2019)” and at: https://doi.org/10.57745/DGOW4Z. The dataset is called “Walnut_Ephesis_export‐2.csv.” The file called “WALNUT_PlantMaterial.tab” allows to link the identifier name of the accessions used in this study with the identifier name of the INRAE walnut germplasm collection. The file called “WATO‐Walnut‐Trait‐Ontology‐V2‐1.csv” gives complete information about the ontology used to phenotype the traits considered in this study.

## References

[tpg270047-bib-0001] Afsharyan, N. P. , Sannemann, W. , Léon, J. , & Ballvora, A. (2020). Effect of epistasis and environment on flowering time in barley reveals a novel flowering‐delaying QTL allele. Journal of Experimental Botany, 71(3), 893–906. 10.1093/jxb/erz477 31781747 PMC6977191

[tpg270047-bib-0002] Ahmadi, Z. , Ghafouri‐Kesbi, F. , & Zamani, P. (2021). Assessing the performance of a novel method for genomic selection: RrBLUP‐method6. Journal of Genetics, 100, Article 24. 10.1007/s12041-021-01275-5 34187971

[tpg270047-bib-0003] Akdemir, D. (2017). *STPGA: Selection of training populations by genetic algorithm* (R package Version 5.2.1) [Computer software]. https://cran.r‐project.org/web/packages/STPGA/STPGA.pdf

[tpg270047-bib-0004] Akpunar, B. E. , Orman, E. , Yagmur, B. , Tanyolac, M. B. , & Ates, D. (2024). Identification of SNP markers linked to calcium and phosphorus accumulation in walnut (*Juglans regia* L.) fruit by GWAS. Scientia Horticulturae, 335, 113341. 10.1016/j.scienta.2024.113341

[tpg270047-bib-0005] Alemu, A. , Åstrand, J. , Montesinos‐López, O. A. , Isidro y Sánchez, J. , Fernández‐Gónzalez, J. , Tadesse, W. , Vetukuri, R. R. , Carlsson, A. S. , Ceplitis, A. , Crossa, J. , Ortiz, R. , & Chawade, A. (2024). Genomic selection in plant breeding: Key factors shaping two decades of progress. Molecular Plant, 17(4), 552–578. 10.1016/j.molp.2024.03.007 38475993

[tpg270047-bib-0006] Amaral, J. S. , Alves, M. R. , Seabra, R. M. , & Oliveira, B. P. P. (2005). Vitamin E composition of walnuts (*Juglans regia* L.): A 3‐year comparative study of different cultivars. Journal of Agricultural and Food Chemistry, 53(13), 5467–5472. 10.1021/jf050342u 15969535

[tpg270047-bib-0007] Arab, M. M. , Marrano, A. , Abdollahi‐Arpanahi, R. , Leslie, C. A. , Askari, H. , Neale, D. B. , & Vahdati, K. (2019). Genome‐wide patterns of population structure and association mapping of nut‐related traits in Persian walnut populations from Iran using the Axiom *J. regia* 700K SNP array. Scientific Reports, 9, Article 6376. 10.1038/s41598-019-42940-1 31015545 PMC6478883

[tpg270047-bib-0008] Bates, D. , Mächler, M. , Bolker, B. , & Walker, S. (2015). Fitting linear mixed‐effects models using lme4. Journal of Statistical Software, 67(1), 1–48. 10.18637/jss.v067.i01

[tpg270047-bib-0009] Ben Hassen, M. , Cao, T. V. , Bartholomé, J. , Orasen, G. , Colombi, C. , Rakotomalala, J. , Razafinimpiasa, L. , Bertone, C. , Biselli, C. , Volante, A. , Desiderio, F. , Jacquin, L. , Valè, G. , & Ahmadi, N. (2018). Rice diversity panel provides accurate genomic predictions for complex traits in the progenies of biparental crosses involving members of the panel. Theoretical and Applied Genetics [Theoretische Und Angewandte Genetik], 131, 417–435. 10.1007/s00122-017-3011-4 29138904 PMC5787227

[tpg270047-bib-0010] Bernard, A. , Barreneche, T. , Delmas, M. , Durand, S. , Pommier, C. , Lheureux, F. , Tranchand, E. , Naudin, M. , & Dirlewanger, E. (2019). The walnut genetic resources of INRA: Chronological phenotypic data and ontology. BMC Research Notes, 12, Article 662. 10.1186/s13104-019-4678-1 31623654 PMC6798330

[tpg270047-bib-0011] Bernard, A. , Crabier, J. , Donkpegan, A. S. L. , Marrano, A. , Lheureux, F. , & Dirlewanger, E. (2021). Genome‐wide association study reveals candidate genes involved in fruit trait variation in Persian walnut (*Juglans regia* L.). Frontiers in Plant Science, 11, Article 607213. 10.3389/fpls.2020.607213 33584750 PMC7873874

[tpg270047-bib-0012] Bernard, A. , Lheureux, F. , & Dirlewanger, E. (2018). Walnut: Past and future of genetic improvement. Tree Genetics & Genomes, 14(1), Article 1. 10.1007/s11295-017-1214-0

[tpg270047-bib-0013] Bernard, A. , Marrano, A. , Donkpegan, A. , Brown, P. J. , Leslie, C. A. , Neale, D. B. , Lheureux, F. , & Dirlewanger, E. (2020). Association and linkage mapping to unravel genetic architecture of phenological traits and lateral bearing in Persian walnut (*Juglans regia* L.). BMC Genomics, 21, Article 203. 10.1186/s12864-020-6616-y 32131731 PMC7057608

[tpg270047-bib-0014] Biscarini, F. , Nazzicari, N. , Bink, M. , Arús, P. , Aranzana, M. J. , Verde, I. , Micali, S. , Pascal, T. , Quilot‐Turion, B. , Lambert, P. , Da Silva Linge, C. , Pacheco, I. , Bassi, D. , Stella, A. , & Rossini, L. (2017). Genome‐enabled predictions for fruit weight and quality from repeated records in European peach progenies. BMC Genomics, 18, Article 432. 10.1186/s12864-017-3781-8 28583089 PMC5460546

[tpg270047-bib-0015] Brault, C. , Doligez, A. , Cunff, L. E. , Coupel‐Ledru, A. , Simonneau, T. , Chiquet, J. , This, P. , & Flutre, T. (2021). Harnessing multivariate, penalized regression methods for genomic prediction and QTL detection of drought‐related traits in grapevine. G3 Genes|Genomes|Genetics, 11(9), jkab248. 10.1093/g3journal/jkab248 34544146 PMC8496232

[tpg270047-bib-0016] Brault, C. , Segura, V. , Roques, M. , Lamblin, P. , Bouckenooghe, V. , Pouzalgues, N. , Cunty, C. , Breil, M. , Frouin, M. , Garcin, L. , Camps, L. , Ducasse, M.‐A. , Romieu, C. , Masson, G. , Julliard, S. , Flutre, T. , & Le Cunff, L. (2024). Enhancing grapevine breeding efficiency through genomic prediction and selection index. G3 Genes|Genomes|Genetics, 14(4), jkae038. 10.1093/g3journal/jkae038 38401528 PMC10989862

[tpg270047-bib-0017] Brault, C. , Segura, V. , This, P. , Le Cunff, L. , Flutre, T. , François, P. , Pons, T. , Péros, J. P. , & Doligez, A. (2022). Across‐population genomic prediction in grapevine opens up promising prospects for breeding. Horticulture Research, 9, uhac041. 10.1093/hr/uhac041 35184162 PMC9070645

[tpg270047-bib-0018] Bükücü, Ş. B. , Sütyemez, M. , Kefayati, S. , Paizila, A. , Jighly, A. , & Kafkas, S. (2020). Major QTL with pleiotropic effects controlling time of leaf budburst and flowering‐related traits in walnut (*Juglans regia* L.). Scientific Reports, 10, Article 15207. 10.1038/s41598-020-71809-x 32938965 PMC7495441

[tpg270047-bib-0019] Burstin, J. , Salloignon, P. , Chabert‐Martinello, M. , Magnin‐Robert, J.‐B. , Siol, M. , Jacquin, F. , Chauveau, A. , Pont, C. , Aubert, G. , Delaitre, C. , Truntzer, C. , & Duc, G. (2015). Genetic diversity and trait genomic prediction in a pea diversity panel. BMC Genomics, 16, Article 105. 10.1186/s12864-015-1266-1 25765216 PMC4355348

[tpg270047-bib-0020] Carrion, J. S. , & Sanchez‐Gomez, P. (1992). Palynological data in support of the survival of walnut (*Juglans regia* L.) in the western Mediterranean area during last glacial times. Journal of Biogeography, 19, 623–630. 10.2307/2845705

[tpg270047-bib-0021] Cazenave, X. , Petit, B. , Lateur, M. , Nybom, H. , Sedlak, J. , Tartarini, S. , Laurens, F. , Durel, C. E. , & Muranty, H. (2022). Combining genetic resources and elite material populations to improve the accuracy of genomic prediction in apple. G3 Genes|Genomes|Genetics, 12(3), jkab420. 10.1093/g3journal/jkab420 34893831 PMC9210277

[tpg270047-bib-0022] Cosmulescu, S. , & Botu, M. (2012). Walnut biodiversity in south‐western Romania‐resource for perspective cultivars. Pakistan Journal of Botany, 44, 307–311.

[tpg270047-bib-0023] Desta, Z. A. , & Ortiz, R. (2014). Genomic selection: Genome‐wide prediction in plant improvement. Trends in Plant Science, 19, 592–601. 10.1016/j.tplants.2014.05.006 24970707

[tpg270047-bib-0024] Ebrahimi, A. , Khadivi‐Khub, A. , Nosrati, Z. , & Karimi, R. (2015). Identification of superior walnut (*Juglans regia* L.) genotypes with late leafing and high kernel quality in Iran. Scientia Horticulturae, 193, 195–201. 10.1016/j.scienta.2015.06.049

[tpg270047-bib-0025] Endelman, J. B. (2011). Ridge regression and other kernels for genomic selection with R package rrBLUP. The Plant Genome, 4, 250–255. 10.3835/plantgenome2011.08.0024

[tpg270047-bib-0026] Fallah, M. , Paizila, A. , Karcı, H. , Arab, M. M. , Sarikhani, S. , Suprun, I. , Rasouli, M. , Hassani, D. , Kafkas, S. , & Vahdati, K. (2024). Validation and implementation of marker‐assisted selection (MAS) for the leafing date trait in Persian walnut populations from Iran. Euphytica, 220, Article 25. 10.1007/s10681-023-03281-3

[tpg270047-bib-0027] Fernández‐González, J. , Akdemir, D. , & Isidro y Sánchez, J. A. (2023). Comparison of methods for training population optimization in genomic selection. Theoretical and Applied Genetics [Theoretische Und Angewandte Genetik], 136(3), Article 30. 10.1007/s00122-023-04265-6 36892603 PMC9998580

[tpg270047-bib-0028] Flutre, T. , Le Cunff, L. , Fodor, A. , Launay, A. , Romieu, C. , Berger, G. , Bertrand, Y. , Terrier, N. , Beccavin, I. , Bouckenooghe, V. , Roques, M. , Pinasseau, L. , Verbaere, A. , Sommerer, N. , Cheynier, V. , Bacilieri, R. , Boursiquot, J. M. , Lacombe, T. , Laucou, V. , … Doligez, A. (2022). A genome‐wide association and prediction study in grapevine deciphers the genetic architecture of multiple traits and identifies genes under many new QTLs. G3 Genes|Genomes|Genetics, 12(7), jkac103. 10.1093/g3journal/jkac103 35485948 PMC9258538

[tpg270047-bib-0029] Forde, H. I. (1975). Walnuts. In J. Janick , & J. N. Moore (Eds.), Advances in fruit breeding (pp. 439–455). Purdue University Press.

[tpg270047-bib-0030] Fugeray‐Scarbel, A. , Bastien, C. , Dupont‐Nivet, M. , & Lemarié, S. , R2D2 Consortium . (2021). Why and how to switch to genomic selection: Lessons from plant and animal breeding experience. Frontiers in Genetics, 9(12), Article 629737. 10.3389/fgene.2021.629737 PMC8301370 34305998

[tpg270047-bib-0031] Ghasemi, M. , Arzani, K. , & Hassani, D. (2012). Evaluation and identification of walnut (*Juglans regia* L.) genotypes in Markazi province of Iran. Crop Breeding Journal, 2, 119–124. 10.22092/cbj.2012.100429

[tpg270047-bib-0032] Gianola, D. , de los Campos, G. , Hill, W. G. , Manfredi, E. , & Fernando, R. (2009). Additive genetic variability and the Bayesian alphabet. Genetics, 183(1), 347–363. 10.1534/genetics.109.103952 19620397 PMC2746159

[tpg270047-bib-0033] Hardner, C. M. , Fikere, M. , Gasic, K. , Da Silva Linge, C. , Worthington, M. , Byrne, D. , Rawandoozi, Z. , & Peace, C. (2022). Multi‐environment genomic prediction for soluble solids content in peach (*Prunus persica*). Frontiers in Plant Science, 13, 960449. 10.3389/fpls.2022.960449 36275520 PMC9583944

[tpg270047-bib-0034] Heslot, N. , Yang, H. P. , Sorrells, M. E. , & Jannink, J. L. (2012). Genomic selection in plant breeding: A comparison of models. Crop Breeding & Genetic, 52, 146–160. 10.2135/cropsci2011.06.0297

[tpg270047-bib-0035] Hoerl, A. E. , & Kennard, R. W. (2000). Ridge regression: Biased estimation for nonorthogonal problems. Technometrics, 42, 80–86. 10.2307/1271436

[tpg270047-bib-0036] Hong, J. P. , Ro, N. , Lee, H. Y. , Kim, G. W. , Kwon, J. K. , Yamamoto, E. , & Kang, B. C. (2020). Genomic selection for prediction of fruit‐related traits in pepper (*Capsicum* spp.). Frontiers in Plant Science, 11, Article 570871. 10.3389/fpls.2020.570871 33193503 PMC7655793

[tpg270047-bib-0037] Imai, A. , Kuniga, T. , Yoshioka, T. , Nonaka, K. , Mitani, N. , Fukamachi, H. , Hiehata, N. , Yamamoto, M. , & Hayashi, T. (2019). Single‐step genomic prediction of fruit‐quality traits using phenotypic records of non‐genotyped relatives in citrus. PLoS ONE, 14(8), e0221880. 10.1371/journal.pone.0221880 31465502 PMC6715226

[tpg270047-bib-0038] Isidro, J. , Jannink, J.‐L. , Akdemir, D. , Poland, J. , Heslot, N. , & Sorrells, M. E. (2015). Training set optimization under population structure in genomic selection. Theoretical and Applied Genetics [Theoretische Und Angewandte Genetik], 128, 145–158. 10.1007/s00122-014-2418-4 25367380 PMC4282691

[tpg270047-bib-0039] Isidro y Sánchez, J. , & Akdemir, D. (2021). Training set optimization for sparse phenotyping in genomic selection: A conceptual overview. Frontiers in Plant Science, 12, Article 715910. 10.3389/fpls.2021.715910 34589099 PMC8475495

[tpg270047-bib-0040] Iwata, H. , Minamikawa, M. F. , Kajiya‐Kanegae, H. , Ishimori, M. , & Hayashi, T. (2016). Genomics‐assisted breeding in fruit trees. Breeding Science, 66(1), 100–115. 10.1270/jsbbs.66.100 27069395 PMC4780794

[tpg270047-bib-0041] Kefayati, S. , Ikhsan, A. S. , Sutyemez, M. , Paizila, A. , Topçu, H. , Bükücü, Ş. B. , & Kafkas, S. (2019). First simple sequence repeat‐based genetic linkage map reveals a major QTL for leafing time in walnut (*Juglans regia* L.). Tree Genetics & Genomes, 15, Article 13. 10.1007/s11295-019-1318-9

[tpg270047-bib-0042] Khadivi‐Khub, A. , Ebrahimi, A. , Mohammadi, A. , & Kari, A. (2015). Characterization and selection of walnut (*Juglans regia* L.) genotypes from seedling origin trees. Tree Genetics & Genomes, 11, Article 54. 10.1007/s11295-015-0882-x

[tpg270047-bib-0043] Kim, G. W. , Hong, J. P. , Lee, H. Y. , Kwon, J. K. , Kim, D. A. , & Kang, B. C. (2022). Genomic selection with fixed‐effect markers improves the prediction accuracy for Capsaicinoid contents in *Capsicum annuum* . Horticulture Research, 9, uhac204. 10.1093/hr/uhac204 36467271 PMC9714256

[tpg270047-bib-0044] Kim, K. H. , Kim, J. Y. , Lim, W. J. , Jeong, S. , Lee, H. Y. , Cho, Y. , Moon, J. K. , & Kim, N. (2020). Genome‐wide association and epistatic interactions of flowering time in soybean cultivar. PLoS ONE, 15(1), e0228114. 10.1371/journal.pone.0228114 31968016 PMC6975553

[tpg270047-bib-0045] Kumar, S. , Chagné, D. , Bink, M. , Volz, R. K. , Whitworth, C. , & Carlisle, C. (2012). Genomic selection for fruit quality traits in apple (*Malus*×*domestica* Borkh.). PLoS ONE, 7(5), e36674. 10.1371/journal.pone.0036674 22574211 PMC3344927

[tpg270047-bib-0046] Kumar, S. , Kirk, C. , Deng, C. H. , Shirtliff, A. , Wiedow, C. , Qin, M. , Wu, J. , & Brewer, L. (2019). Marker‐trait associations and genomic predictions of interspecific pear (*Pyrus*) fruit characteristics. Scientific Reports, 9(1), Article 9072. 10.1038/s41598-019-45618-w 31227781 PMC6588632

[tpg270047-bib-0047] Laloë, D. (1993). Precision and information in linear models of genetic evaluation. Genetics Selection Evolution, 25(6), 557–576. 10.1186/1297-9686-25-6-557

[tpg270047-bib-0048] Liu, C. , Tu, Y. , Liao, S. , Fu, X. , Lian, X. , He, Y. , Xie, W. , & Wang, G. (2021). Genome‐wide association study of flowering time reveals complex genetic heterogeneity and epistatic interactions in rice. Gene, 770, 145353. 10.1016/j.gene.2020.145353 33333227

[tpg270047-bib-0049] Liu, X. , Huang, M. , Fan, B. , Buckler, E. S. , & Zhang, Z. (2016). Iterative usage of fixed and random effect models for powerful and efficient genome‐wide association studies. PLoS Genetics, 12(2), e1005767. 10.1371/journal.pgen.1005767 26828793 PMC4734661

[tpg270047-bib-0050] Liu, X. , Wang, H. , Wang, H. , Guo, Z. , Xu, X. , Liu, J. , Wang, S. , Li, W. , Zou, C. , Prasanna, B. M. , Olsen, M. , Huang, C. , & Xu, Y. (2018). Factors affecting genomic selection revealed by empirical evidence in maize. The Crop Journal, 6(4), 341–352. 10.1016/j.cj.2018.03.005

[tpg270047-bib-0051] Luedeling, E. , & Gassner, A. (2012). Partial least squares regression for analyzing walnut phenology in California. Agricultural and Forest Meteorology, 158–159, 43–52. 10.1016/j.agrformet.2011.10.020

[tpg270047-bib-0052] Makowsky, R. , Pajewski, N. M. , Klimentidis, Y. C. , Vazquez, A. I. , Duarte, C. W. , Allison, D. B. , & de Los Campos, G. (2011). Beyond missing heritability: Prediction of complex traits. PLoS Genetics, 7(4), e1002051. 10.1371/journal.pgen.1002051 21552331 PMC3084207

[tpg270047-bib-0053] Manning, W. E. (1978). The classification within the Juglandaceae. Annals of the Missouri Botanical Garden, 65, 1058–1087. 10.2307/2398782

[tpg270047-bib-0054] Marrano, A. , Martínez‐García, P. J. , Bianco, L. , Sideli, G. M. , Di Pierro, E. A. , Leslie, C. A. , Stevens, K. A. , Crepeau, M. W. , Troggio, M. , Langley, C. H. , & Neale, D. B. (2019). A new genomic tool for walnut (*Juglans regia* L.): Development and validation of the high‐density Axiom™ *J. regia* 700K SNP genotyping array. Plant Biotechnology Journal, 17, 1027–1036. 10.1111/pbi.13034 30515952 PMC6523593

[tpg270047-bib-0055] Marrano, A. , Sideli, G. M. , Leslie, C. A. , Cheng, H. , & Neale, D. B. (2019). Deciphering of the genetic control of phenology, yield, and pellicle color in Persian walnut (*Juglans regia* L.). Frontiers in Plant Science, 10, Article 1140. 10.3389/fpls.2019.01140 31616449 PMC6764078

[tpg270047-bib-0056] Martínez‐García, P. J. , Crepeau, M. W. , Puiu, D. , Gonzalez‐Ibeas, D. , Whalen, J. , Stevens, K. A. , Paul, R. , Butterfield, T. S. , Britton, M. T. , Reagan, R. L. , Chakraborty, S. , Walawage, S. L. , Vasquez‐Gross, H. A. , Cardeno, C. , Famula, R. A. , Pratt, K. , Kuruganti, S. , Aradhya, M. K. , Leslie, C. A. , … Neale, D. B. (2016). The walnut (*Juglans regia*) genome sequence reveals diversity in genes coding for the biosynthesis of non‐structural polyphenols. Plant Journal, 87, 507–532. 10.1111/tpj.13207 27145194

[tpg270047-bib-0057] Meuwissen, T. H. E. , Hayes, B. J. , & Goddard, M. E. (2001). Prediction of total genetic value using genome‐wide dense marker maps. Genetics, 157, 1819–1829. 10.1093/genetics/157.4.1819 11290733 PMC1461589

[tpg270047-bib-0058] Minamikawa, M. F. , Kunihisa, M. , Moriya, S. , Shimizu, T. , Inamori, M. , & Iwata, H. (2024). Genomic prediction and genome‐wide association study using combined genotypic data from different genotyping systems: Application to apple fruit quality traits. Horticulture Research, 11, uhae131. 10.1093/hr/uhae131 38979105 PMC11228094

[tpg270047-bib-0059] Minamikawa, M. F. , Nonaka, K. , Kaminuma, E. , Kajiya‐Kanegae, H. , Onogi, A. , Goto, S. , Yoshioka, T. , Imai, A. , Hamada, H. , Hayashi, T. , Matsumoto, S. , Katayose, Y. , Toyoda, A. , Fujiyama, A. , Nakamura, Y. , Shimizu, T. , & Iwata, H. (2017). Genome‐wide association study and genomic prediction in citrus: Potential of genomics‐assisted breeding for fruit quality traits. Scientific Reports, 7(1), Article 4721. 10.1038/s41598-017-05100-x 28680114 PMC5498537

[tpg270047-bib-0060] Muir, W. M. (2007). Comparison of genomic and traditional BLUP‐estimated breeding value accuracy and selection response under alternative trait and genomic parameters. Journal of Animal Breeding and Genetics, 124, 342–355. 10.1111/j.1439-0388.2007.00700.x 18076471

[tpg270047-bib-0061] Munyengwa, N. , Peace, C. , Dillon, N. L. , Ortiz‐Barrientos, D. , Christie, N. , Myburg, A. A. , & Hardner, C. (2023). SNP and haplotype‐based genomic prediction of fruit quality traits in sweet cherry (*Prunus avium*). Acta Horticulturae, 1362, 173–180. 10.17660/ActaHortic.2023.1362.23

[tpg270047-bib-0062] Nsibi, M. , Gouble, B. , Bureau, S. , Flutre, T. , Sauvage, C. , Audergon, J. M. , & Regnard, J. L. (2020). Adoption and optimization of genomic selection to sustain breeding for apricot fruit quality. G3 Genes|Genomes|Genetics, 10(12), 4513–4529. 10.1534/g3.120.401452 33067307 PMC7718743

[tpg270047-bib-0063] Pérez, P. , & de los Campos, G. (2014). Genome‐wide regression and prediction with the BGLR statistical package. Genetics, 198(2), 483–495. 10.1534/genetics.114.164442 25009151 PMC4196607

[tpg270047-bib-0064] Poland, J. A. , Brown, P. J. , Sorrells, M. E. , & Jannink, J. L. (2012). Development of high‐density genetic maps for barley and wheat using a novel two‐enzyme genotyping‐by‐sequencing approach. PLoS ONE, 7, e32253. 10.1371/journal.pone.0032253 22389690 PMC3289635

[tpg270047-bib-0065] Pollegioni, P. , Woeste, K. , Chiocchini, F. , Del Lungo, S. , Ciolfi, M. , Olimpieri, I. , Tortolano, V. , Clark, J. O , Hemery, G. E. , Mapelli, S. , & Malvolti, M. E. (2017). Rethinking the history of common walnut (*Juglans regia* L.) in Europe: Its origins and human interactions. PLoS ONE, 12(3), e0172541. 10.1371/journal.pone.0172541 28257470 PMC5336217

[tpg270047-bib-0066] Rehder, A. (1947). Manual of cultivated trees and shrubs . The Macmillan Company.

[tpg270047-bib-0067] Resende, M. F. R. , Muñoz, P. , Resende, M. D. V. , Garrick, D. J. , Fernando, R. L. , Davis, J. M. , Jokela, E. J. , Martin, T. A. , Peter, G. F. , & Kirst, M. (2012). Accuracy of genomic selection methods in a standard data set of loblolly pine (*Pinus taeda* L.). Genetics, 190, 1503–1510. 10.1534/genetics.111.137026 22271763 PMC3316659

[tpg270047-bib-0068] Rincent, R. , Laloë, D. , Nicolas, S. , Altmann, T. , Brunel, D. , Revilla, P. , Rodríguez, V. M. , Moreno‐Gonzalez, J. , Melchinger, A. , Bauer, E. , Schoen, C. C. , Meyer, N. , Giauffret, C. , Bauland, C. , Jamin, P. , Laborde, J. , Monod, H. , Flament, P. , Charcosset, A. , & Moreau, L. (2012). Maximizing the reliability of genomic selection by optimizing the calibration set of reference individuals: Comparison of methods in two diverse groups of maize inbreds (*Zea mays* L.). Genetics, 192(2), 715–728. 10.1534/genetics.112.141473 22865733 PMC3454892

[tpg270047-bib-0069] Roth, M. , Muranty, H. , Di Guardo, M. , Guerra, W. , Patocchi, A. , & Costa, F. (2020). Genomic prediction of fruit texture and training population optimization towards the application of genomic selection in apple. Horticulture Research, 7, 148. 10.1038/s41438-020-00370-5 32922820 PMC7459338

[tpg270047-bib-0070] Roux‐Cuvelier, M. , Grisoni, M. , Bellec, A. , Bloquel, E. , Charron, C. , Delalande, M. , Delmas, M. , Didier, A. , Durel, C.‐E. , Duval, C.‐H. , Esnault, F. , Feugey, L. , Geoffriau, E. , Khadari, B. , Lepers‐Andrzejewski, S. , Luro, F. , Marchal, C. , Pernet, A. , Salinier, J. , ….. Kahane, R. (2021). Conservation of horticultural genetic resources in France. Chronica Horticulturae, 61(2), 21–36.

[tpg270047-bib-0071] Sarinelli, J. M. , Murphy, J. P. , Tyagi, P. , Holland, J. B. , Johnson, J. W. , Mergoum, M. , Mason, R. E. , Babar, A. , Harrison, S. , Sutton, R. , Griffey, C. A. , & Brown‐Guedira, G. (2019). Training population selection and use of fixed effects to optimize genomic predictions in a historical USA winter wheat panel. Theoretical and Applied Genetics [Theoretische Und Angewandte Genetik], 132, 1247–1261. 10.1007/s00122-019-03276-6 30680419 PMC6449317

[tpg270047-bib-0072] Şen, S. M. , & Karadeniz, T. (2015). The nutritional value of walnut. Journal of Hygienic Engineering and Design, 11, 68–71.

[tpg270047-bib-0073] Shah, R. A. , Bakshi, P. , Sharma, N. , Jasrotia, A. , Itoo, H. , Gupta, R. , & Singh, A. (2021). Diversity assessment and selection of superior Persian walnut (*Juglans regia* L.) trees of seedling origin from North‐Western Himalayan region. Resources, Environment and Sustainability, 3, 100015. 10.1016/j.resenv.2021.100015

[tpg270047-bib-0074] Sharma, R. M. , Kour, K. , Singh, B. , Yadav, S. , Kotwal, N. , Rana, J. C. , & Anand, R. (2014). Selection and characterization of elite walnut (*Juglans regia* L.) clone from seedling origin trees in North Western Himalayan region of India. Australian Journal of Crop Science, 8, 257–262.

[tpg270047-bib-0075] Sideli, G. M. , Mcatee, P. , Marrano, A. , Allen, B. J. , Brown, P. J. , Butterfield, T. S. , Dandekar, A. M. , Leslie, C. A. , & Neale, D. B. (2020). Genetic analysis of walnut (*Juglans regia* L.) pellicle pigment variation through a novel, high‐throughput phenotyping platform. G3 Genes|Genomes|Genetics, 10(12), 4411–4424. 10.1534/g3.120.401580 33008832 PMC7718756

[tpg270047-bib-0076] Sun, M. , Zhang, M. , Kumar, S. , Qin, M. , Liu, Y. , Wang, R. , Qi, K. , Zhang, S. , Chang, W. , Li, J. , & Wu, J. (2024). Genomic selection of eight fruit traits in pear. Horticultural Plant Journal, 10(2), 318–326. 10.1016/j.hpj.2023.04.008

[tpg270047-bib-0077] VanRaden, P. M. (2008). Efficient methods to compute genomic predictions. Journal of Dairy Science, 91(11), 4414–4423. 10.3168/jds.2007-0980 18946147

[tpg270047-bib-0078] Wang, J. , Ye, H. , Zhou, H. , Chen, P. , Liu, H. , Xi, R. , Wang, G. , Hou, N. , & Zhao, P. (2022). Genome‐wide association analysis of 101 accessions dissects the genetic basis of shell thickness for genetic improvement in Persian walnut (*Juglans regia* L.). BMC Plant Biology, 22, Article 436. 10.1186/s12870-022-03824-1 36096735 PMC9469530

[tpg270047-bib-0079] Woodworth, R. H. (1930). Meiosis of micro‐sporogenesis within the Juglandaceae. American Journal of Botany, 17, 863–869. 10.2307/2435868

[tpg270047-bib-0080] Zeven, A. C. , & Zhukovsky, P. M. (1975). Dictionary of cultivated plants and their centres of diversity excluding ornamentals, forest trees, and lower plants. Centre for Agricultural Publishing and Documentation.

